# Microenvironment drug resistance in multiple myeloma: emerging new players

**DOI:** 10.18632/oncotarget.10849

**Published:** 2016-07-26

**Authors:** Lucia Di Marzo, Vanessa Desantis, Antonio Giovanni Solimando, Simona Ruggieri, Tiziana Annese, Beatrice Nico, Ruggiero Fumarulo, Angelo Vacca, Maria Antonia Frassanito

**Affiliations:** ^1^ Department of Biomedical Sciences and Human Oncology, Internal Medicine Section, University of Bari Medical School, Bari, Italy; ^2^ Department of Basic Medical Sciences, Neurosciences and Sensory Organs, University of Bari Medical School, Bari, Italy; ^3^ Department of Biomedical Sciences and Human Oncology, General Pathology Section, Bari, Italy

**Keywords:** cancer-associated fibroblasts, drug resistance, exosomes, microRNAs, multiple myeloma

## Abstract

Multiple myeloma (MM) drug resistance (DR) is a multistep transformation process based on a powerful interplay between bone marrow stromal cells and MM cells that allows the latter to escape anti-myeloma therapies. Here we present an overview of the role of the bone marrow microenvironment in both soluble factors-mediated drug resistance (SFM-DR) and cell adhesion-mediated drug resistance (CAM-DR), focusing on the role of new players, namely miRNAs, exosomes and cancer-associated fibroblasts.

## DRUG RESISTANCE

Multiple myeloma (MM) is the second most common hematologic malignancy characterized by the infiltration of monoclonal malignant plasma cells (MM cells) at multiple sites within the bone marrow (BM) compartment [1]. The pathophysiology of MM depends both on several oncogenic events occurring in MM cells, i.e. genomic/chromosomal instability, gene mutations and chromosomal translocations, and on extracellular factors, i.e. dynamic interactions between MM cells and the BM microenvironment (BMME) in a reciprocal pro-survival loop [1]. Despite the significantly improved response rate and overall survival of MM patients since the advent of novel agents such as bortezomib, thalidomide, lenalidomide, and autologous stem cell transplantation, MM remains an incurable malignancy with a 5-years survival rate of around 40% [2]. Indeed, the majority of patients relapse or become refractory to therapies, implying that drug resistance (DR) prevents effective treatment of MM.

Resistance to chemotherapy can be *acquired* or *de novo* DR [3]. *Acquired* resistance develops gradually as a result of sequential genetic and epigenetic changes that ultimately confer the tumor cells a complex drug-resistant phenotype. Acquired resistance depends on multifactorial processes, namely decreased drug uptake, expression of new drug-efflux pumps, drug metabolism, repair of DNA damage, alterations of cell proliferation and/or apoptosis [4]. It is usually studied *in vitro via* prolonged exposure of cells to a cytotoxic agent until the drug resistant phenotype is acquired. *De novo* resistance is present before the drug exposure and then selected during the drug treatment [3]. Recent studies have demonstrated, by inter-phase fluorescence *in situ* hybridization (iFISH) [5] and flow cytometry [6], the heterogeneity of MM cells at genetic (chromosome number, genetic translocations and mutations), clonal and cell differentiation levels. In particular, flow cytometry analysis reveals the presence of multiple cell clones in patients at diagnosis that are selected by *in vivo* therapeutic pressure and induce a distinct phenotypic MM cell subclone with different clonogenic and cytogenetic profiles in minimal residual disease [6]. A form of d*e novo* resistance is environment-mediated drug resistance (EMDR), in which the BMME protects tumor cells from chemotherapy, radiotherapy or receptor-targeting drugs [7]. Overall, these observations highlight that MM progression and drug resistance are multistep transformation processes regulated by a complex cross-talk between MM cells and the BMME.

Here we show the latest findings on EMDR in MM. In particular, we focus on the role of microRNAs (miRNAs/miRs), exosomes and cancer-associated fibroblasts (CAFs) as new BMME players contributing to EMDR.

## THE BMME AS A NICHE FOR MM CELLS

**Figure 1:**
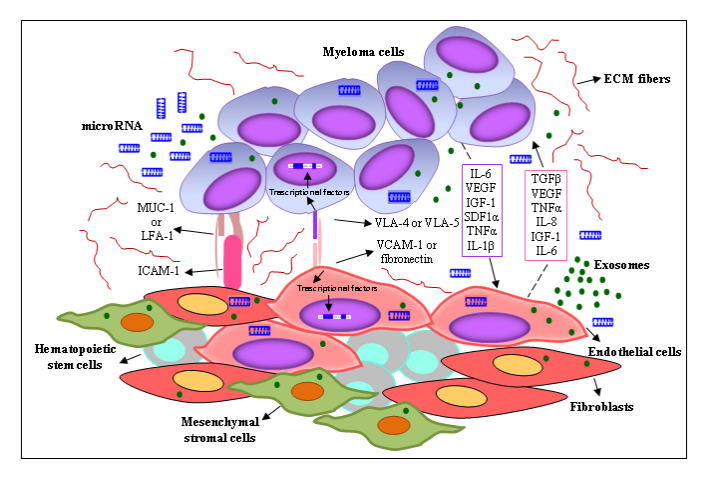
Interplay between MM cells and the surrounding microenvironment MM cells are surrounded by a complex BMME composed of ECM proteins and several cell types, including BM stromal cells (ECs, mesenchymal stromal cells, CAFs). The cross-talk between MM cells and BM stromal cells is regulated by different mechanisms: (*i*) cell-to-cell adhesion between MM cells and ECM components/BM stromal cells; and (*ii*) soluble factors, i.e. cytokines, chemokines, growth factors, exosomes and miRNAs released by the BM stromal cells and MM cells, with autocrine and paracrine effects. Both mechanisms activate several signaling pathways in BM stromal cells and tumor cells, leading to MM drug resistance.

The BMME includes a non-cellular compartment formed by extracellular matrix (ECM) proteins (laminin, fibronectin and collagen) and soluble factors (cytokines, growth factors, chemokines), and a rich cellular compartment constituted by hematopoietic cells (myeloid cells, T lymphocytes, B lymphocytes, NK cells) and non-hematopoietic cells (fibroblasts, osteoblasts, osteoclasts, endothelial cells (ECs), endothelial progenitor cells (EPCs), pericytes, mesenchymal stem cells, mesenchymal stromal cells) (Figure 1). All these cells form specialized microenvironment niches, the osteoblast/endosteal and vascular niches, that play a key role in MM cell growth, survival and DR [8]. The osteoblast niche is located in the endosteum, at the interface between trabecular bone and BM, and regulates hematopoietic stem cell quiescence and self-renewal, hence hematopoiesis. The blood vessel-rich vascular niche controls stem cell mobilization, proliferation and differentiation [9]. The osteoblast and vascular niches are adjacent and show a mutually related secretion of several cytokines/growth factors and/or expression of adhesion molecules, creating a permissive microenvironment in the BM, namely “MM niches” [9]. MM cells home to and reside in these niches where they are protected from apoptotic stimuli and acquire the DR phenotype. In the niches, MM cells suppress osteoblastic cells, leading to impaired bone formation and the development of osteolytic lesions, and enhance angiogenesis *via* angiogenic factors secreted by MM cells, ECs, and BM stromal cells, thus promoting disease progression. The inability of conventional anti-cancer drugs to cure MM has reinforced EMDR. During chemotherapy, the interactions of a small subset of tumor cells with the BMME allow them to survive in a quiescent and protected state, resulting in minimal residual disease that progressively develops the acquired-resistance phenotype [10]. Using intravital two-photon microscopy in live mice, Lawson *et al*. [11] demonstrated that a small subpopulation of MM cells colonizes BM sites close to collagen-expressing osteoblasts or bone-lining cells, and falls into a dormant state by down-regulating the expression of genes that govern the cell cycle. Dormant MM cells are resistant to melphalan; however, cells may be activated by microenvironmental signals that can switch cancer cell dormancy “on” or “off”. McMillin *et al.* [12] developed a tumor cell-specific *in vitro* bioluminescence imaging assay that analyzes the effect of drugs on tumor cell viability in the presence and absence of BM stromal cells. They identified a stroma-induced signature in tumor cells, including AKT, Ras, NF-kB, HIF-1α, Myc, hTert and IRF4 signaling pathways, which is correlated with an adverse clinical prognosis.

EMDR can be subdivided into: *i)* soluble factors-mediated resistance (SFM-DR), which relies on cytokines, chemokines and growth factors, and *ii)* cell adhesion-mediated resistance (CAM-DR) resulting from adhesion of tumor cells to BM stromal cells or to ECM components.

## SOLUBLE FACTORS-MEDIATED DRUG RESISTANCE

The BMME contains soluble factors secreted by BM stromal cells or MM cells themselves, that play a multifaceted role in both MM onset and progression, and the development of DR. Since soluble factors can act on several cell targets, they regulate multiple processes such as cell growth and apoptosis, cell migration, adhesion, and angiogenesis. Notably, soluble factors cooperate among themselves through a complex communication network, creating the permissive BM niche.

The most important growth and survival factor is IL-6. It is secreted by both BM stromal cells and MM cells and inhibits MM apoptosis induced by serum starvation, dexamethasone and Fas ligand [13, 14]. Autocrine IL-6 production is associated with a malignant MM cells phenotype, i.e. a high proliferative index and resistance to dexamethasone-induced apoptosis [15]. By binding with its receptor, IL-6 triggers activation of the RAS/Raf/MEK/MAPK, JAK/STAT3 and PI3K/Akt signaling pathways [16]. Activation of the JAK/STAT3 pathway protects tumor cells from Fas-mediated apoptosis by upregulating the anti-apoptotic proteins Mcl-1, Bcl-X_L_ and c-Myc. The IL-6/STAT3 signaling pathway is tightly controlled by the SOCS-1 protein, a negative regulator of cytokine signaling that is frequently inactivated by hypermethylation in MM patients, resulting in an uncontrolled IL-6 signaling [17]. Secretion of IL-6 by BM stromal cells is up-regulated by many molecules/cytokines (IL-1β, CD40, TNF-α, VEGF, TGF-β) and by MM cell adhesion *via* activation of the NF-kB pathway [8]. A major cytokine responsible for paracrine IL-6 production is the IL-1β secreted by MM cells, that increases the expression of adhesion molecules, stimulating IL-6 secretion by BM stromal cells [18]. All these data emphasize the key role of IL-6 in MM and explain why IL-1β and IL-6 may be useful targets in MM treatment [18]. However, several *in vitro* preclinical studies targeting the IL-6 signaling pathway, including antibodies against IL-6 and IL-6 receptors (CNTO328), IL-6 antisense oligonucleotides and IL-6 super antagonist Sant7, were unsuccessful due to autocrine IL-6 production, and/or activation of the IL-6/STAT3 and NF-κB pathways following MM cells-BM stromal cells interactions [19].

IL-6, in turn, enhances the secretion of VEGF, the most important angiogenic factor [20]. Angiogenesis is a constant hallmark in MM progression, and correlates with tumor growth, relapse and DR [21]. It is regulated by a balance between angiogenic and antiangiogenic molecules but this is lost in the angiogenic switch [20] that occurs during MM progression. The levels of VEGF, produced by both MM cells and BM stromal cells, increase during MM progression. FGF-2, TGF-β, and TNFα also stimulate its secretion [22]. By binding with its receptors, VEGF-R1 and VEGF-R2, VEGF stimulates vascular permeability, ECs proliferation, migration, invasion into surrounding tissue, and whole capillary tube formation [23]. It also governs the differentiation and survival of BM stromal cells [22]. Antivascular and antiangiogenic agents, including bortezomib and thalidomide, have cytotoxic effects on MM cells and ECs by inhibiting the secretion of multiple angiogenic factors including VEGF, HGF and FGF-2 [24, 25].

HGF is upregulated during MM progression. It enhances the expression of its receptor, cMET, and of other angiogenic factors, including VEGF and FGF-2, and suppresses the secretion of thrombospondin 1, an endogenous angiogenesis inhibitor [26]. The HGF/cMET pathway is involved in the MM pathogenesis: it is constitutively activated in MM cells [27] and ECs [28] from relapsed and resistant patients, and mediates DR [27, 28]. In ECs, the HGF/cMET loop enhances the expression of VEGF-A/VEGF-R2, sustaining MM angiogenesis [27]. *In vitro* and *in vivo* studies of a novel selective cMET inhibitor, SU11274, tested alone or in combination with bortezomib, lenalidomide and dexamethasone, suggest that the HGF/cMET pathway may be envisaged as a new therapeutic target for relapsed and refractory MM patients [28].

IGF-1 is produced by both MM cells and BM stromal cells, and contributes to MM pathobiology, inducing MM cell growth, survival, migration, MM-associated angiogenesis and osteolysis [29]. By binding its receptor, IGF-1R, it activates the PI3K/Akt and MEK/ERK signaling pathways, that induce overexpression of the anti-apoptotic proteins Bcl-X_L_, Bcl-2 and downregulation of the pro-apoptotic protein Bim [29, 30]. IGF-1R is overexpressed on MM cells: this aberrant expression associated with high IGF-1 levels has been related to disease progression and poor prognosis [29, 31]. *In vitro* and *in vivo* studies show that an increased expression and activation of the IGF-1/IGF-1R pathway in MM cells is associated with resistance to bortezomib [32]. Based on these observations, several therapeutic agents targeting the IGF-1/IGF-1R pathway have been developed and analyzed in preclinical studies [29]. Menu *et al.* [33, 34] demonstrated that picropodophyllin, a non-ATP-competitive IGF-1R tyrosine kinase inhibitor, inhibits tumor growth [33], MM-associated angiogenesis and bone disease [34] in 5T33MM-treated mice.

Another important soluble factor is IL-8 (CXCL8), a pro-inflammatory and angiogenic chemokine mainly secreted by BM stromal cells [35]. It contributes to tumor progression by inducing tumor cell proliferation, survival, migration and angiogenesis through the phosphorylation of VEGF-R2 [36, 37]. *In vitro* studies demonstrate that IL-8 contributes to BM stromal cells-induced NF-κB activity in MM cells and the consequent resistance to bortezomib [38].

Finally, the stromal cell-derived factor (SDF-1)/CXCR4 axis plays an important role in cell trafficking within the BM, favoring the formation of pro-metastatic BM niches and DR [39, 40]. SDF-1 (CXCL12) is constitutively expressed and released by BM stromal cells and fibroblasts, while its receptor CXCR4 is expressed by MM cells and ECs [40]. Activation of the SDF-1/CXCR4 axis promotes trans-endothelial migration, BM homing, migration and adhesion of MM cells. BM stromal cells-MM cells adhesion up-regulates SDF-1 secretion that, in turn, increases integrins expression and IL-6 and VEGF secretion, supporting MM cell growth and DR [41]. Using an *in vivo* murine xenografted mouse model, Roccaro *et al*. [42, 43] demonstrated that CXCR4 blockade by the monoclonal antibody Ulocuplumab, as well as neutralization of SDF-1 by Olaptesed-pegol (PEGylated mirror-image l-oligonucleotide) inhibits MM bone-to-bone cell dissemination and hence tumor progression.

**Table 1:** Adhesion molecules involved in MM drug resistance

PROTEINS	LIGAND	FUNCTION	REFs
Integrin β_1_	Laminin, Collagen type-VI, Fibronectin	Cell protection from cell cycle-dependent drug therapies	44, 45
Integrin β_7_	Fibronectin, E-cadherin	Cell adhesion, migration, and homing	44, 46
Integrin α_v_β_3_	Vitronectin, Fibronectin	Cell proliferation, protease secretion, invasion and spreading	44, 47
^1^VLA-4 (α_4_β_1_)	Fibronectin, ^2^VCAM-1	Cell adhesion, migration, homing and invasion, angiogenesis, cytokines secretion	48, 49, 50, 51
^1^VLA-5 (α_5_β_1_)	Fibronectin	Cell homing and migration. Its down-expression correlates with MM progression	44, 49
^2^VCAM- 1	^1^VLA-4	Cells migration, homing and invasion	52
^3^LFA-1	^5^ICAM-1	Cell adhesion, proliferation and survival, angiogenesis, tumor dissemination	48, 50, 53
^4^MUC-1	^5^ICAM-1	Cells adhesion, growth and survival, disease progression	54
CD44 isoforms	Hyaluronan	Cell adhesion and invasion	44, 50
CD138 (syndecan-1)	Fibronectin	Cell adhesion	44

1 VLA = Very Late Activation Antigen

2 VCAM = Vascular Cell Adhesion Molecule

3 LFA = Lymphocyte Function-Associated Antigen

4 MUC = Mucin-1 antigen

5 ICAM = Intercellular Adhesion Molecule.

## CELL ADHESION-MEDIATED DRUG RESISTANCE

CAM-DR is a mechanism whereby MM cells escape the cytotoxic effects of anti-cancer therapy *via* adhesive interactions with BM stromal cells and/or ECM components. It is achieved through adhesion molecules of the integrin family [44-51], CD138 (syndecan-1) [44], CD44 [44, 50], Vascular Cell Adhesion Molecule-1 (VCAM-1) [52], Lymphocyte Function-Associated Antigen-1 (LFA-1) [48, 50, 53], Mucin-1 antigen (MUC-1) [54], and Intercellular Adhesion Molecule-1 (ICAM-1) [48, 50, 53, 54]. Among the integrins, major fibronectin receptors include the Very Late Activation Antigen (VLA)-4 (α_4_β_1_), VLA-5 (α_5_β_1_), α_v_β_3_ and β_7_ integrins, that mediate MM cell trafficking and DR [44]. Damiano *et al*. [49] described, for the first time, CAM-DR as a reversible DR phenotype in fibronectin-adherent MM cells. The adhesive interactions between MM cells and BM stromal cells are complex because they involve several adhesion molecules expressed on both MM cells and BM stromal cells [48, 50-54]. MM cells-BM stromal cells adhesion triggers IL-6 secretion, NF-κB activation in stromal cells [51] and the up-regulation of many signaling pathways resulting in MM cell proliferation and survival [55].

Several studies [49, 51, 55, 56] have described CAM-DR to doxorubicin, melphalan, vincristine, dexamethasone and mitoxantrone in MM cell lines and patients primary MM cells due to their adhesion to fibronectin or BM stromal cells. Inhibition of cell adhesive interactions by shRNA-mediated knockdown of the α_4_ subunit of VLA-4 (CD49d) or by anti-α_4_ antibodies reverses CAM-DR, sensitizing MM cells to drug therapy [56]. VLA-4 is strongly expressed on MM cells and is the only integrin able to mediate both MM cell-ECM and MM cell-BM stromal cells interactions *via* separate binding sites [57]. Bortezomib overcomes CAM-DR to vincristine and dexamethasone by down-regulating VLA-4 expression on MM cells, thus inhibiting MM cell adhesion to fibronectin and BM stromal cells [56]. Other integrins involved in CAM-DR are VLA-5 and β_7_: the former supports cells survival by up-regulating Bcl-2 expression [58], the latter increases MM cells adhesion, migration, and homing into BM, and reduces bortezomib- and melphalan-induced apoptosis [46]. Moreover, β1-integrin-mediated CAM-DR protects cells from cell cycle-dependent drug therapies, such as the topoisomerase inhibitor etoposide, by up-regulating p27^kip1^ and halting the cell cycle [45]. In line with these observations, Paiva *et al*. [59] demonstrated that MM cells in minimal residual disease show an overexpression of integrins and adhesion molecules even compared to the cells at first diagnosis.

MM cells-BM stromal cells interactions are also mediated by Notch [60]. Binding of Notch receptors on MM cells with the specific ligands on BM stromal cells confers mitoxantrone and melphalan resistance. Indeed, Notch activation results in an increasing secretion of IL-6, IGF-1, and VEGF that, as previously described, contribute to create a permissive BMME [8]. Finally, a relevant consequence of MM cells-BM stromal cells adhesion is the IL-6 mediated up-regulation of PD-L1 (CD274 or B7-H1) expression on MM cells, that increases their proliferative ability, induces resistance to dexamethasone and melphalan, and down-regulates anti-tumor immune T cell responses [61].

All these findings demonstrate that CAM-DR influences the therapeutic response and suggest the importance of introducing anti-adhesion strategies in combination with chemotherapeutic drugs. Podar *et al.* [62] evaluated the effect of Natalizumab, a selective adhesion-molecule inhibitor, which binds α_4_ integrins and prevents MM cells interactions with ECM and BM stromal cells. They observed that Natalizumab inhibits MM cell proliferation, VEGF secretion and angiogenesis and enhances the anti-MM activity of bortezomib and dexamethasone, supporting the hypothesis that anti-adhesion treatment could improve the current therapeutic strategies for MM.

## NEW PLAYERS: MIRNAS, EXOSOMES AND CANCER-ASSOCIATED FIBROBLASTS

A new mechanism of intercellular communication between BM stromal cells and MM cells involved in MM pathogenesis and DR is shown to be accomplished by miRNAs, exosomes and CAFs.

### miRNAs

miRNAs are endogenous, single-stranded, non-coding RNAs (19-25 nt), that regulate gene expression by targeting the 3′-untranslated region (3′-UTR) of mRNAs, and therefore inhibit protein translation, regulating a wide range of physiologic and pathologic cellular processes such as proliferation, differentiation, metabolism, aging and cell death [63, 64]. Deregulation of miRNAs has been described in various tumors including MM, where they can function either as tumor suppressors or oncogenes [65, 66].

In MM, a deregulated miRNAs expression in tumor cells has been associated to disease progression, pathobiology and DR, through modulating the expression of target genes involved in several pathways, such as p53, IGF-1/IGF-1R, VEGF/VEGF-R, NF-κB, IL-6-STAT3, SOCS1 [67-71]. In particular, miRNAs deregulation is correlated with MM clinical stages and/or MM molecular subtypes [67-70]. Over-expression of miR-21, -106b∼25 cluster, -181a/b has been observed in MM and MGUS cells compared to healthy plasma cells, while a selective upregulation of miR-32 and -17∼92 cluster has been demonstrated in MM cells and cell lines but not in MGUS cells [67]. Lionetti *et al.* [70], in an integrated analysis of miRNAs expression with genome-wide copy number variations and heterozygosity, noticed a strong correlation between miRNAs expression and IGH translocation and heterozygosity. Seckinger *et al.* [71] investigated miRNAs expression in 92 purified MM and MGUS cells and normal plasma cells using miChip-array, that analyzes 559 human miRNAs. Compared to normal plasma cells, MM cells showed 67 differentially expressed miRNAs, and MGUS cells 20. The authors found no correlation between miRNAs expression and MM molecular classifications. On the contrary, a deregulated expression of miR-135a, -135b, -200a, -200b and -596 was related with overall survival.

Emerging evidence relates miRNAs expression with anti-cancer drug activity or DR [69, 72]. The expression of miR-27a is associated with bortezomib resistance in MM patients [73]. Bortezomib treatment of MM cell lines significantly decreases the expression of miR-27a, whose gene target is the cyclin-dependent kinase 5 (CDK5), a major modulator of bortezomib sensitivity. These findings are in agreement with previous data [74] that demonstrate an increased bortezomib sensitivity of MM cell lines and patients tumor cells by down-regulating CDK5. miR-29 acts as tumor suppressor miRNA in several hematological malignancies [75]. It is down-regulated in patients MM cells [76, 77] and in MM cell lines with acquired resistance to bortezomib, carfilzomib and ixazomib [78]. Amodio *et al.* [76] described a miR-29b-Sp1 loop that explains the role of miR-29b in bortezomib-induced apoptosis. Indeed, Sp1, a transcription factor that regulates the cell cycle and apoptosis-related genes, whose expression is in turn governed by 26S proteasome [79], negatively regulates miR-29b expression. Bortezomib treatment up-regulates miR-29b levels through a down-regulation of Sp1, enhancing drug-induced apoptosis in patients MM cells and in MM cell lines. Enforced expression of miR-29b in MM cells enhances bortezomib-induced apoptosis through the reduction of proteasome activator PA200 levels [78].

Since the BMME plays a central role in determining MM progression and DR [7], the involvement of miRNAs in the cross-talk between the BMME and MM cells has been investigated. Roccaro *et al*. [80] described a MM-specific miRNAs signature in relapsed/refractory MM cells compared to their normal counterpart, characterized by a down-expression or the absence of miRs-15a and -16. MM-BM stromal cells interactions decrease miRs-15a and -16 expression in MM cells, inducing cell survival and IL-6-mediated bortezomib resistance [81, 82]. Enforced expression of pre-miRs-15a and -16 inhibits MM-BM stromal cells adhesion and MM cell proliferation, by inhibiting AKT3, ribosomal-protein-S6, MAP-kinases, NF-κB-activator MAP3KIP3, as well as limiting angiogenesis by decreasing VEGF secretion [80].

Adhesion of MM cells to BM stromal cells up-regulates miR-125a-5p [83] and -21 [84-86] levels in tumor cells. An increased expression of miR125a-5p in MM cells is associated to a subset of MM patients carrying the t(4;14) translocation [70]. Enforced expression of miR-125a mimics can downregulate the p53 pathway-related genes, i.e. TP53, BAX, MDM2, CDKN1A, leading to tumor growth by influencing cell proliferation, migration and apoptosis. In addition, it targets the TNFα-induced protein 3 (TNFAIP3) and activates the NF-κB pathways [87] suggesting that it may cause an aberrant NF-κB activation in bortezomib-resistant MM cells. miR-125a-5p reduces the expression of p53-responsive miR-192 and -194, which enhance cell cycle arrest and apoptosis. Based on these observations, Leotta *et al.* [83] suggested the combined use of miR-125 inhibitors and miR-192 and -194 mimics to treat MM. miR-21 promotes MM cell growth and viability. It is up-regulated following MM-BM stromal cells adhesion *via* the IL-6/STAT3 [84, 85] and NF-κB [86] pathways. MM cells overexpressing miR-21 are resistant to dexamethasone- and doxorubicin-induced apoptosis, and demonstrate a central role of miR-21 in CAM-DR. Targeting miR-21 inhibits MM cell growth, counteracting the protective effect of BM stromal cells and, in combination with dexamethasone and/or doxorubicin, synergistically triggers MM cell apoptosis [86].

miRNAs are secreted in biological fluids (e.g. plasma, serum, saliva, urine) [88] as nuclease resistant entities, packaged with RNA-binding proteins [89] or contained in microvesicles as exosomes (detailed below). Circulating miRNAs are fully functional and able to act as signaling molecules inside the recipient cells by modulating the expression of their target genes. Increasing evidence suggests that miRNAs could be diagnostic and prognostic markers in human tumors, including MM [90, 91]. Rocci *et al.* [91] analyzed the serum miRNAs levels of a large cohort of newly diagnosed MM patients and correlated the miRNAs levels with the clinical outcome to test their prognostic relevance. Among 800 miRNAs, they identified two circulating miR-16 and -25 whose serum levels were associated with overall survival. Nevertheless, they did not observe a correlation between miRNAs serum levels and miRNAs expression in MM cells, implying that circulating miRNAs do not resemble the miRNAs profile of tumor cells in the BM. Different results were illustrated by other authors [92-94]. Wang *et al.* [92] demonstrated a miRNAs signature in the extracellular BMME of MM patients that mirrored serum and plasma circulating miRNAs. A low expression of miR-let-7a, -15a and -106b was observed in the BMME and MM cells. Kubiczkova *et al.* [93] identified 5 circulating miRNAs (miR-34a, -130a, -744, let-7d, let-7e) that were differentially expressed in MM and MGUS serum compared to healthy subjects. The miR-744 and let-7e levels were correlated with overall survival. Navarro *et al.* [94] described a serum miRNAs signature (miR-16, -17, -19b, -20a and -660) as a potential diagnostic and prognostic tool in MM. Low levels of both miR-19b and -331 were shown to be a marker of short progression-free survival after autologous stem-cell transplantation.

Further studies of circulating miRNAs in MM are needed to elucidate their cell origin (cancer or normal, live or apoptotic cells), and their relationship with anti-MM drug activity or DR.

### Exosomes

Exosomes are small membranous vesicles (40-100 nm) released in the extracellular milieu by several cell types [95-97] in physiological and pathological conditions. They mediate local and systemic cell-to-cell communication and regulate cell behavior by transferring mRNA, miRNAs and proteins, through fusion with the cell membrane or through endocytosis followed by internalization of recipient cells [98]. In the MM context, the exosomes-MM cells interaction is mediated by fibronectin binding to heparan sulfate, expressed on the surface of both exosomes and MM cells [99]. This binding activates p38 and pERK signaling and the expression of the downstream target genes DKK1 and MMP-9, and promotes MM progression by inducing tumor cell spread and ECs invasion [100]. The disruption of fibronectin-heparan sulfate interactions blocks exosome binding to MM cells or BM stromal cells, highlighting a specific cross-talk fostered by exosomes in the BMME [100]. The involvement of exosomes released by MM cells and BM stromal cells, as an active vehicle that can modulate the microenvironment and promote tumor progression and DR, has been investigated by *in vitro* and *in vivo* studies [101-103]. Exosomes derived from BM mesenchymal stromal cells of MM patients show a different functional activity compared to that of normal donors: the former facilitate MM progression and spread whereas the latter inhibit MM cell growth. Tissue-engineered bones loaded with MM cells alone, as control, and in the presence of either MM or normal BM-mesenchymal stromal cells-derived exosomes, were subcutaneously injected into SCID mice. Bioluminescence *in vivo* imaging showed a significantly higher tumor growth rate in mice transplanted with MM BM-mesenchymal stromal cells-derived exosomes than in those transplanted with normal BM-MSC-derived exosomes. Using *in vivo* confocal imaging, a higher ability of MM BM-mesenchymal stromal cells-derived exosomes to disseminate to the distant BM niches *in vivo* was demonstrated. These effects were related to a protein cargo of MM BM stromal cells-exosomes, i.e. high levels of IL-6, CCL2, γ-catenin, fibronectin, and to the absence of the tumor suppressor miR-15a [101]. The *in vivo* involvement of exosomes in the MM cells/BM stromal cells cross-talk has been demonstrated in the murine syngeneic 5T33MM model [102, 103]. Tumor exosomes contain multiple angiogenesis-related proteins, such as angiogenin, HGF, MMP-9, serpin E1, tissue inhibitor of metallopeptidase-1, thrombospondin 1 and VEGF, that promote ECs growth and angiogenesis. Furthermore, MM cells exosomes induce the growth of myeloid-derived suppressor cells and enhance their immunosuppressive capacity *in vivo* by up-regulating inducible nitric oxide synthase [102]. In turn, BM stromal cells-exosomes modulate the proliferation, survival, migration and bortezomib resistance of MM cells [103]. These effects are related to the exosome proteins content, activating several survival pathways, including c-Jun N-terminal kinase, p38, p53, AKT, and inhibiting MM cells bortezomib-induced apoptosis through the modulation of Bcl-2, caspase-9, -3 and PARP expression [103].

The exosome secretion, content and functionality depend on the tumor BMME and tumor phenotype. Umezu *et al*. [104] demonstrated that MM cells under hypoxia conditions produce more exosomes than the parental cells under normoxia, and that their content and function are different. Exosomes derived from hypoxic MM cells contain miR-135b whose target gene is the hypoxia-inducible factor-1α (HIF-1α) subunit inhibitor (FIH-1). Transfer of exosomal miR-135b to ECs reduces the expression of FIH-1 and increases HIF-1 transcriptional activity, accelerating angiogenesis both *in vitro* and *in vivo*. These observations may explain the HIF-1α protein expression and stabilization described in BM ECs from patients with relapsed/refractory MM [105].

Finally, higher exosomes levels have been observed in body fluids of cancer patients compared to healthy subjects, suggesting that exosomes could be used as biomarkers in the diagnosis and prognosis of several tumors including MM [106-109].

## CANCER-ASSOCIATED FIBROBLASTS

CAFs are the most populous cell type within the tumor microenvironment of many solid [110, 111] and hematological malignancies [112]. In MM patients BM, the CAFs population increases, and parallels the clinical stages [113]. According to literature [114], the BM CAFs exhibit phenotypic similarities to myofibroblasts or activated fibroblasts. They are CD45^-^ cells expressing α-smooth muscle actin (α-SMA), fibroblast-specific protein-1 (FSP-1), fibroblast activation protein (FAP), and other markers as platelet-derived growth factor receptor α and β (PDGFR α/β), neuron-glial antigen2 (NG2), CD31, CD144, VEGF-R2, CD33, CD146 and CD90 that are specific for different cell types. This suggests that CAFs may derive from multiple cell lineages: resident fibroblasts, mesenchymal stem cells, *via* the mesenchymal transition [115], ECs and hematopoietic stem and progenitor cells, *via* the endothelial-mesenchymal transition [116].

*In vitro* and i*n vivo* experiments highlight a mutual interplay between CAFs and tumor cells during MM onset and progression. MM cells induce and maintain the CAFs-activated phenotype, their proliferation and recruitment *via* TGF-β [113]. CAFs, in turn, modify the BM stroma and influence chemotaxis, adhesion, proliferation, and apoptosis of MM cells through cell-to-cell contact involving β_3_, β_7_, VLA-4, VLA-5 and α_V_β_3_ integrins expressed on MM cells and β_3_ and β_7_ integrins expressed on CAFs, and the secretion of TGF-β, HGF, IGF-1, IL-1, IL-6 and SDF-1α by both cell types [113].

**Figure 2:**
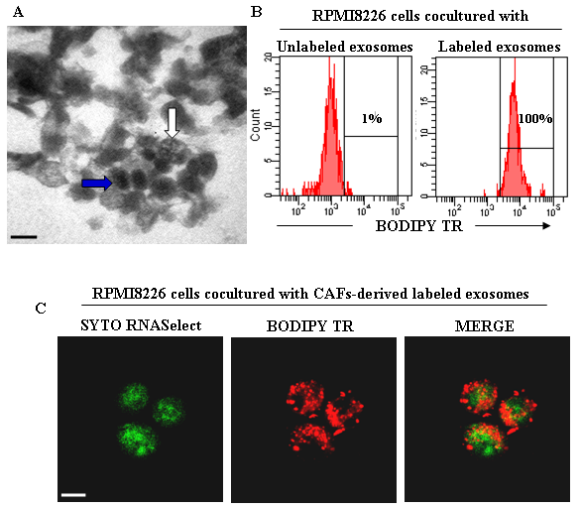
CAFs-derived exosomes and their uptake from MM cells **A.** Transmission electron microscopy of exosomes isolated from BM CAFs of MM patients showing heterogeneous features of vesicles with an electrondense core (blue arrow) and multivesicular body (white arrow). Scale bar, 0.2 μm. **B.** Flow cytometry analysis of exosomes uptake by RPMI8226 cells. The RPMI8226 cells were co-cultured with unlabeled and BODIPY TR ceramide-labeled CAFs-derived exosomes. **C.** Confocal dual immunofluorescence images of RPMI8226 cells swallowed CAFs-derived exosomes labeled with SYTO RNASelect (green) and BODIPY TR ceramide (red), specific for RNAs and cell membranes, respectively. Scale bar, 7.5 μm.

Recently, we demonstrated that BM CAFs from bortezomib-resistant patients are resistant *in vitro* to the drug and prevent the bortezomib-induced apoptosis of co-cultured MM cells [117]. This protection depends on the ability of bortezomib to foster bortezomib-resistant CAFs to secrete several anti-apoptotic cytokine/growth factors (as previously described), such as IGF-1, IL-6, IL-8, TGF-β, and exosomes. Preliminary data demonstrate a release of exosomes from bortezomib-treated CAFs that are swallowed by MM cells (Figure 2), thus preventing their bortezomib-induced apoptosis (unpublished data).

Proteomic and phospho-proteomic analyses reveal that the bortezomib-DR of MM CAFs is associated to cellular stress and activation of pro-survival autophagy mediated by the autocrine TGF-β pathway [117]. Indeed, blockade of the TGF-β pathway by a TβR-I/II inhibitor induces apoptosis of bortezomib-resistant CAFs, by inhibiting the Smad2/3 and autophagy signaling pathways, and overcomes bortezomib resistance of MM cells conferred by CAFs [117]. Therefore, CAFs are emerging as a novel potentially therapeutic target in MM by means of various strategies [112, 118] that act on both MM cells and CAFs or on CAFs alone. In particular, Öhlund *et al*. [118] identified four possible strategies to target the pro-tumorigenic effects of CAFs. These strategies include targeting the stromal barrier in order to increase the drugs delivery; the inhibition of CAFs-secreted factors that promote tumor progression and DR; the depletion or blockage of the ECM components and/or integrins, and, finally, the targeting of CAFs by deactivating the CAFs phenotype to that of quiescent, normal fibroblasts.

## CONCLUSIONS

A large volume of research in MM has highlighted the cardinal role of the BMME as a complex signaling molecules network, in which MM onset, progression and DR are regulated by a contact-dependent and -independent interplay between MM cells and their surrounding microenvironment. The role of the BMME network has been seen to be more complex since the discovery of the new players, miRNAs, exosomes and CAFs. The therapeutic failure of novel MM agents (first and second generation proteasome inhibitors, IMIDs, etc.) that target cell adhesion, cytokines secretion and survival pathways may be explained by their involvement. Notably, CAFs, miRNAs deregulation and/or the exosomes cargo (miRNAs/cytokines/proteins) could permit MM cells to achieve apoptotic escape and/or prosurvival autophagy by modulating alternative signaling pathways. Nevertheless, in-depth investigations are needed to better elucidate the role of miRNAs, exosomes and CAFs in affecting a tumor-prone BM niche.

In conclusion, MM drug resistance, being a multistep transformation process in which several players cooperate among themselves, provides the rationale for a multiple targets therapeutic approach with the aim of creating an unsupportive BMME and thereby enabling anti-MM therapies.
